# Ion Currents Mediated by TRPA1 Channels in Freshly Dissociated Rat Articular Chondrocytes: Biophysical Properties and Regulation by Inflammatory Processes

**DOI:** 10.3390/ph18030332

**Published:** 2025-02-26

**Authors:** Arturo Ponce, Lidia Jimenez, Maria Luisa Roldan, Liora Shoshani

**Affiliations:** Department of Physiology, Biophysics and Neurosciences, CINVESTAV-IPN, Mexico City 07360, Mexico; lidia.jimenez@cinvestav.mx (L.J.); luisa.roldan@cinvestav.mx (M.L.R.); shoshani@cinvestav.mx (L.S.)

**Keywords:** cartilage, articular chondrocytes, inflammation, TRPA1 channels, whole-cell patch-clamp, AITC, IL-1α, IL-1β, LPS

## Abstract

**Background**: Articular chondrocytes are specialized cells in synovial joint cartilage, responsible for maintaining and regenerating the extracellular matrix. Inflammation disrupts the balance between matrix synthesis and degradation, leading to cartilage breakdown. This process, commonly observed in conditions such as osteoarthritis, results in chondrocyte dysfunction and accelerates joint degeneration. Since TRPA1 channels are implicated in inflammatory processes, this study investigates the expression of TRPA1 channels in freshly dissociated rat articular chondrocytes and their modulation by anti-inflammatory agents. **Methods**: We used the whole-cell patch-clamp method to assess TRPA1 channel expression and modulation. **Results**: Freshly dissociated chondrocytes exhibit ion currents attributable to TRPA1 channel expression, with higher magnitudes observed in medium-sized cells. These currents decrease over time in primary culture. Treatment with pro-inflammatory agents (IL-1α, IL-1β, and LPS) increases TRPA1′s current magnitude. IL-1β treatment directly induces transient TRPA1 currents. Several signaling components activated during inflammation contribute to the IL-1β-induced enhancement of TRPA1 current density, including IL-1 R1, the adaptor protein MyD88, and the downstream kinases IRAK1 and IRAK4. **Conclusions**: Our findings demonstrate that healthy rat chondrocytes express functional TRPA1 channels and that inflammatory processes modulate their expression.

## 1. Introduction

Articular cartilage is a smooth, flexible tissue that covers the ends of bones within joints, facilitating low-friction movement and even distribution of mechanical forces. It is primarily composed of water, collagen fibers, and proteoglycans, which provide strength and elasticity to withstand repeated stress. This specialized tissue lacks blood vessels, nerves, and lymphatic vessels, relying on synovial fluid for nutrient delivery and waste removal. Due to its avascular nature, articular cartilage has a limited capacity for healing and regeneration, making it susceptible to degeneration and damage over time [1,2,3,4].

Articular chondrocytes (AChs) are specialized cells embedded within the extracellular matrix of articular cartilage, playing a crucial role in maintaining its structure and function. These cells synthesize and regulate key matrix components, such as collagen and proteoglycans, which contribute to the cartilage’s strength and flexibility [5,6]. Although metabolically active, AChs are confined within isolated lacunae and rely on nutrient diffusion from synovial fluid due to the absence of a direct blood supply. They originate from mesenchymal cells [7] and are distributed across distinct cartilage layers, exhibiting variations in size and differentiation depending on their location [8].

Dynamic compression of articular chondrocytes activates molecular mechanisms that support cartilage health. However, prolonged static or excessive mechanical compression can accelerate cartilage degeneration, highlighting the link between abnormal mechanical loading and the development of osteoarthritis [9,10].

Cartilage inflammation occurs when the tissue becomes swollen, painful, and tender due to immune responses or injury. This condition can result from trauma, infections, autoimmune disorders, or chronic diseases such as osteoarthritis [11,12]. Because cartilage lacks blood vessels, its ability to fight infection and repair itself is limited, making it more vulnerable to prolonged inflammation and damage. Chronic inflammation can degrade the cartilage matrix, impairing function and potentially leading to joint stiffness [13] and osteoarthritis progression [14].

Articular chondrocytes express a diverse range of ion channels [15,16], including voltage-gated sodium (Na^+^) channels [17], voltage-gated potassium (K^+^) channels [18,19,20,21,22], calcium-dependent K^+^ channels [23], voltage-gated calcium channels [24], TRPV channels [25], aquaporins [26], and voltage-gated Cl− channels [27]. Various mechanosensitive channels have also been identified [28,29,30], including piezo 1 and 2 [31], TRPV4 [22,32], swelling-activated chloride channels (TMEM16) [33], and TREK-1 [34].

Transient receptor potential (TRP) channels are a diverse group of ion channels located in cell membranes that detect environmental stimuli such as temperature, pressure, and chemical signals. These channels regulate calcium and sodium ion flow, influencing processes such as pain perception, thermoregulation, and cell signaling. Based on amino acid similarities, TRP channels are classified into six families: TRPC, TRPM, TRPV, TRPA, TRPP, and TRPML [35,36,37].

The transient receptor potential ankyrin 1 (TRPA1) channel, the sole mammalian member of the TRPA family, serves as a molecular sensor for chemical irritants, environmental stimuli, and oxidative stress. It is activated by various harmful substances, including allyl isothiocyanate (found in mustard) [38,39]. TRPA1 channels have attracted significant interest due to their involvement in pathological processes such as pain and inflammation across various tissues [40,41,42,43]. However, the expression and specific role of TRPA1 channels in the molecular physiology of chondrocytes—whether in healthy or diseased states—remain poorly understood.

In this study, we used electrophysiological methods to demonstrate that freshly dissociated chondrocytes from healthy rats exhibit ionic currents associated with TRPA1 channel activity. We found that TRPA1 expression correlates with cell size and becomes dysregulated over time under primary culture conditions. Additionally, we investigated whether exposure to pro-inflammatory agents, including IL-1α, IL-1β, and lipopolysaccharide (LPS), influences TRPA1 channel expression.

## 2. Results

### 2.1. Freshly Isolated Rat Articular Chondrocytes Exhibit Currents Associated with TRPA1 Channel Expression

To determine whether rat articular chondrocytes generate currents linked to TRPA1 channels, we conducted whole-cell patch-clamp experiments on freshly dissociated cells.

#### 2.1.1. AITC Induces a Transient Current in Freshly Dissociated Chondrocytes

**Figure 1:**
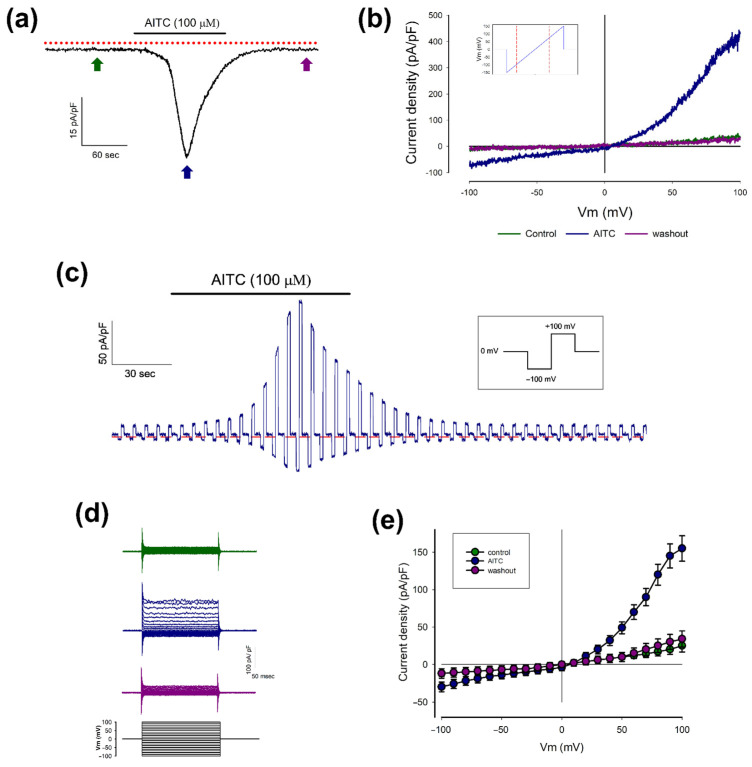
Biophysical characteristics of AITC-induced currents: (**a**) A representative current trace shows the effect of adding AITC (100 μM) to the external medium. This trace was recorded from a freshly dissociated chondrocyte while holding the voltage at −70 mV. The dotted red line indicates zero current. (**b**) Graph displays representative current traces obtained from ramp-like stimulation (as shown in the inset) before, during, and after the addition of AITC to the extracellular medium of freshly dissociated chondrocytes, as indicated by the arrows in (**a**). (**c**) Continuous trace of current recorded from a freshly dissociated chondrocyte in response to a stimulation protocol consisting of square voltage pulses. The pulses alternate between −100 mV and +100 mV from a holding voltage of 0 mV, as indicated in the inset. (**d**) Series of current traces obtained after stimulation of chondrocytes with a protocol of square pulses of voltage before (green), during (blue), and after (cherry) the addition of AITC to the extracellular medium. (**e**) Relationship between the average current density (±S.E., n = 12) and the stimulation voltage of each of the episodes. The three different plots were obtained before, during, and after the addition of AITC to the extracellular medium.

First, we examined the effect of allyl isothiocyanate (AITC), a naturally occurring organic compound found in mustard, horseradish, and wasabi, known to activate TRPA1 channels in various cell types [44]. As shown in Figure 1a, under whole-cell patch-clamp conditions, the addition of AITC (100 μM) to the extracellular medium while holding the membrane potential at −70 mV induced a transient inward current. This current began increasing shortly after AITC application, reached a peak, and then declined despite the continued presence of AITC.

Figure 1b presents three representative current traces recorded in response to ramp-like voltage stimulation, captured before (control), during (AITC), and after AITC application (washout). Both before and after AITC exposure, ramp-like stimulation produced minimal currents. However, shortly after AITC was introduced into the medium, an outwardly rectified current with a reversal potential near 0 mV was observed.

Figure 1c displays a continuous current trace generated by alternating square pulse stimulations of −100 mV and +100 mV, applied intermittently before, during, and after AITC addition and washout. Like the pattern described in Figure 1a, AITC application led to a gradual increase in current values at both +100 mV and −100 mV. Notably, the absolute magnitude of the current at +100 mV was significantly greater than at −100 mV, consistent with the outward rectification profile observed in Figure 1b.

Figure 1d presents current traces recorded before (green), during (blue), and after (purple) AITC exposure, in response to a series of square pulses ranging from −100 mV to +100 mV in 10 mV increments, starting from a holding potential of 0 mV. Figure 1e illustrates three plots showing the average current density (±S.E.) obtained from 12 freshly dissociated chondrocytes as a function of membrane potential. The shape of these plots aligns with the current traces recorded under ramp-like stimulation, as shown in Figure 1b.

Collectively, these results indicate that freshly dissociated healthy rat chondrocytes exhibit currents consistent with TRPA1 channel activity (I-TRPA1).

#### 2.1.2. Pharmacological Characteristics of Currents Mediated by TRPA1

To further validate our findings, we examined the effects of additional compounds known to act specifically as TRPA1 agonists or antagonists. Alongside AITC, these included the included: (1) PF-4840154, a pyrimidine-derived compound identified as a selective agonist for both rat and human TRPA1 channels [45]; (2) JT010, a chloroacetyl-group-containing compound that exhibits high selectivity for TRPA1, with an EC50 of less than 1 nM [46]; (3) HC-030031, a selective TRPA1 blocker that inhibits TRPA1-mediated calcium influx triggered by AITC and formalin [47,48,49]; and (4) TCS-5861528, a selective TRPA1 blocker that inhibits TRPA1-mediated calcium influx induced by AITC and 4-hydroxynonenal [50,51].

For each compound, we performed whole-cell patch-clamp experiments on freshly dissociated chondrocytes to assess their effects at various concentrations and determine the EC50. For the agonists (AITC, PF-4840154, and JT010), we conducted multiple trials at each concentration, measuring the peak current density (dIpeak) induced by adding the compound to the extracellular medium. For the antagonists, we measured dIpeak triggered by AITC (30 μM), which was applied to the extracellular medium three minutes after pre-incubating the cells with the antagonist (either TCS-5861528 or HC-030031) at the designated concentration.

**Figure 2:**
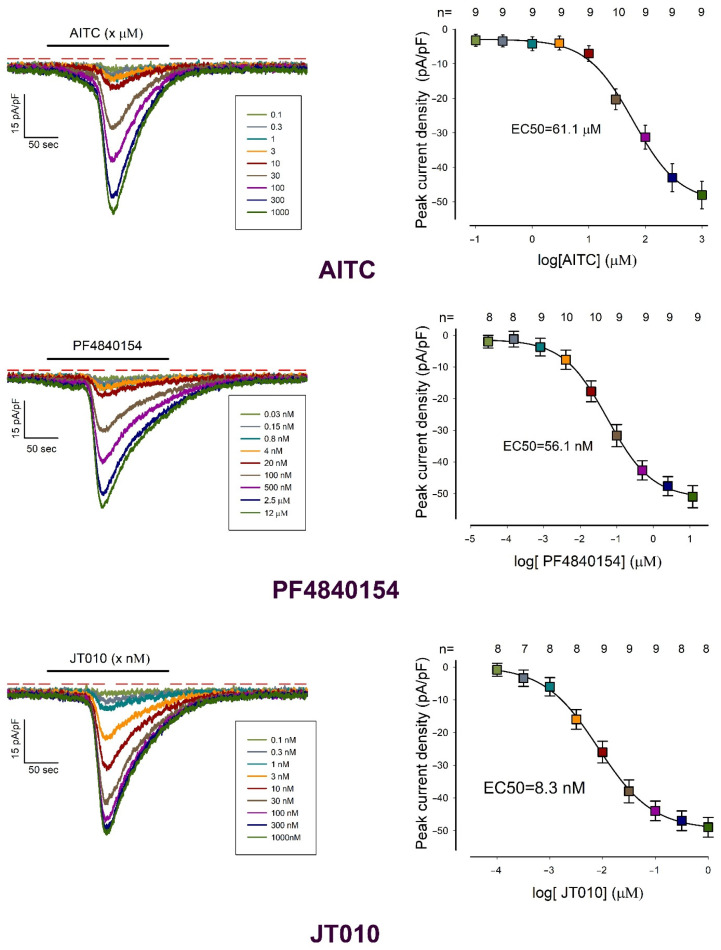
TRPA1 channel-specific agonists induce transient currents in newly dissociated rat chondrocytes. Each panel shows, on the left side, a series of representative current traces describing the effect of TRPA1 agonists AITC (**top**), PF4810154 (**middle2**) and JT10 (**bottom**) at a range of concentrations; on the right side is shown a graph describing the relationship between the average value (±S.E.) of the peak current density and the log of the concentration of each agonist. The colors of each current density value match those of the representative strokes on the left side.

**Figure 3:**
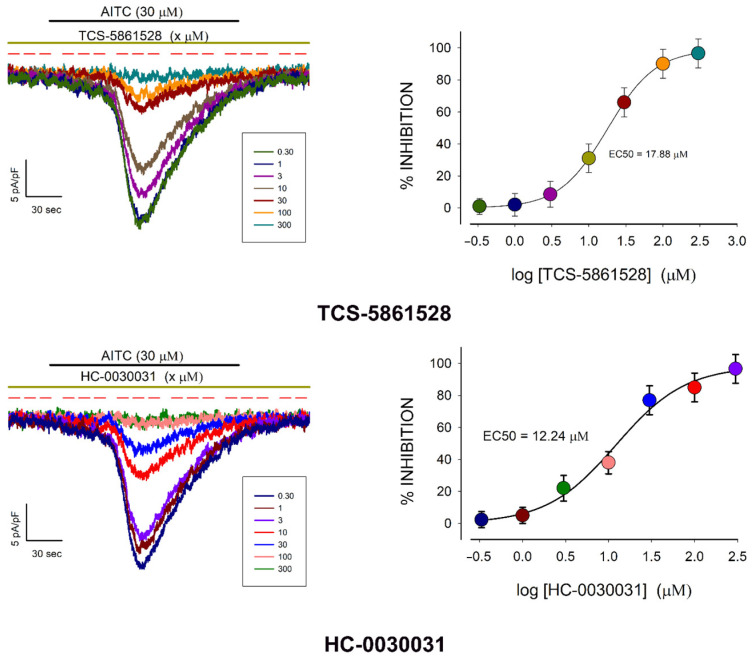
TRPA1 channel-specific antagonists abolish AITC-induced transient currents in freshly dissociated rat chondrocytes. Each panel shows, on the left side, a series of representative current traces describing the effect of TRPA1-channel antagonists TCS-5861528 (**top**) and HC-0030031 (**bottom**) at various increasing concentrations; on the right side is shown a graph describing the relationship between the average value (±S.E.) of the peak current density and the log of the concentration of each agonist. The colors of each current density value match those of the representative strokes on the left side.

Figure 2 and Figure 3 present the results for the agonists and antagonists, respectively. In both figures, each compound’s effects are depicted in two panels: the left panel displays representative current traces recorded at various concentrations, while the right panel shows a plot correlating the average peak current density (±S.E.) with the logarithm of the tested concentration. The EC50 values were estimated by fitting the data to a sigmoidal curve, as described in Section 4.5.1.

The three agonists elicited similar responses, characterized by the onset of a transient current that gradually increased following a latency period, peaked, and then slowly declined despite the continued presence of the agonist. While the time to reach the peak response was comparable among the three agonists, the rate of deactivation varied: PF-4840154 exhibited the slowest deactivation, whereas AITC deactivated more rapidly. Additionally, the three compounds displayed significantly different sensitivities, as indicated by their respective adjusted EC50 values.

The observation that the two TRPA1-selective agonists, PF-4840154 and JT010, induced transient currents resembling those elicited by AITC strongly supports the conclusion that freshly dissociated rat chondrocytes exhibit functional TRPA1-mediated currents. This conclusion is further corroborated by the inhibition of AITC-induced currents by the two TRPA1-specific antagonists, TCS-5861528 and HC-030031, as shown in Figure 3. Moreover, the near-complete inhibition of AITC-induced currents by these antagonists strongly suggests that the observed currents are mediated by TRPA1 channels, ruling out the involvement of other channels that may be activated by AITC. Consequently, the peak current density observed upon the addition of 30 μM AITC serves as a reliable indicator of TRPA1 channel density in chondrocytes. In the subsequent assays described below, this parameter is referred to as pId-TRPA1.

### 2.2. In Freshly Isolated Chondrocytes, the Magnitude of pId-TRPA1 Varies with Cell Size

Articular cartilage is composed of three distinct zones or layers—superficial, intermediate, and deep—each characterized by a unique extracellular matrix composition. The chondrocytes within these layers vary in size, shape, and function. Specifically, chondrocytes in the superficial layer are significantly smaller than those in the intermediate and deep layers, with cells in the deep layer being larger than those in the intermediate layer [2,52,53,54].

This observation prompted us to investigate whether TRPA1 channel expression in chondrocytes is associated with cell size. To explore this, we measured pId-TRPA1 in freshly isolated chondrocytes, categorizing them into small, medium, or large groups based on their membrane capacitance. A one-way analysis of variance (ANOVA) was then performed, followed by pairwise comparisons among the three groups, to test the hypothesis that pId-TRPA1 (as an estimator of TRPA1 channel density) is related to cell size.

**Figure 4:**
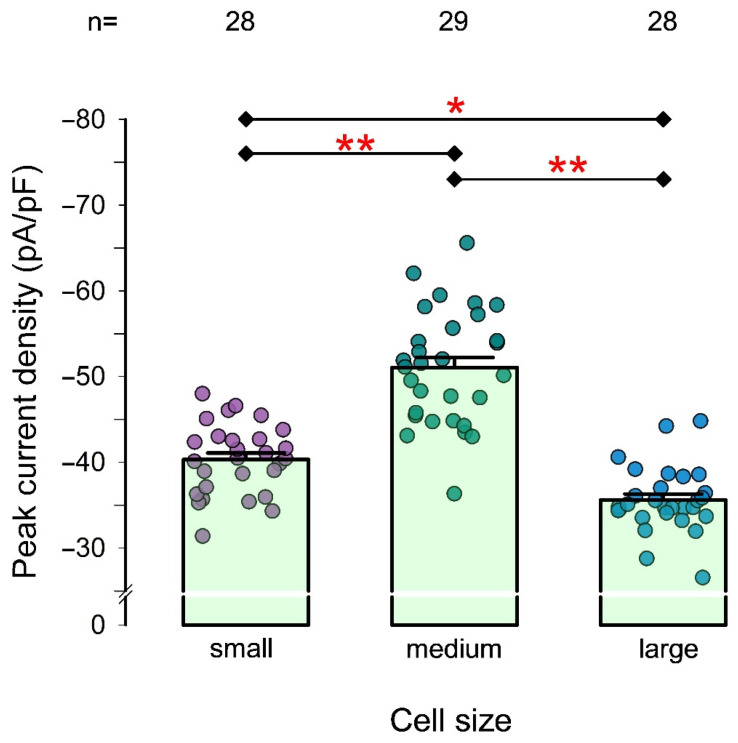
The magnitude of pId-TRPA1 of freshly dissociated articular chondrocytes depends on their size. Bar chart compares the average value (±S.E.) of pId-TRPA1 measured in freshly dissociated chondrocytes classified as either small, medium, or large. On each bar, the dots identify individual measurements. The numbers above the bars indicate the number of chondrocytes tested in each group. Asterisks above the lines connecting two different bars indicate a statistically significant difference. (*) denotes *p* < 0.01; (**) denotes *p* < 0.001 after a *t*-test.

As shown in Figure 4, the mean pId-TRPA1 values (± S.E.) for the small, medium, and large groups were −40.3 ± 0.78 (n = 28), −51.0 ± 1.212 (n = 29), and −35.6 ± 0.70 (n = 28), respectively. ANOVA revealed a statistically significant relationship between cell size and pId-TRPA1 (*p* < 0.001). Pairwise comparisons confirmed significant differences among all categories, with medium-sized cells exhibiting a substantially higher average pId-TRPA1 value compared to both the small and large groups.

### 2.3. In Chondrocytes in Primary Culture, the Magnitude of pId-TRPA1 Gradually Decreases over Time

**Figure 5:**
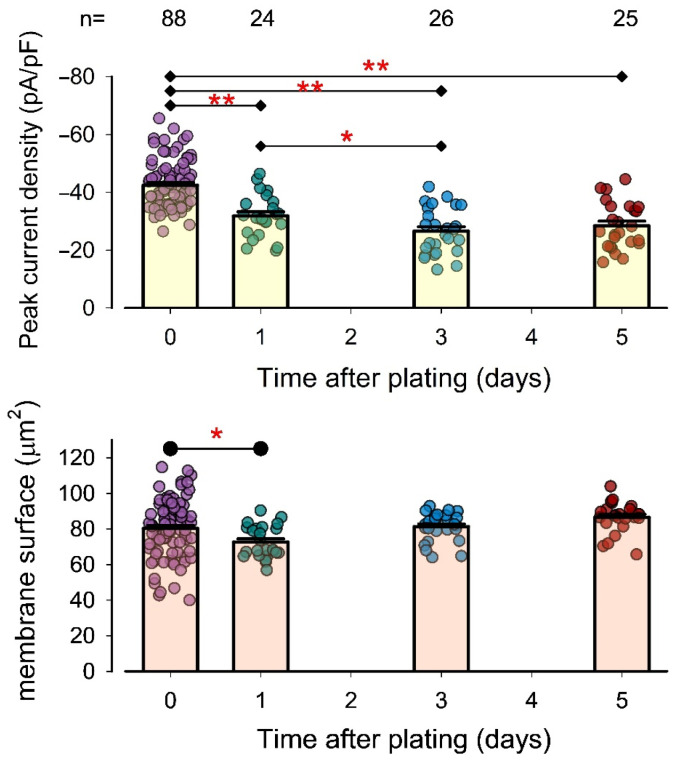
The density of TRPA1 channels decreases over time in the primary culture. Bar graphs compare the average value (±S.E.) of pId-TRPA1 (**above**) and the membrane surface (**below**) from chondrocytes freshly dissociated (time zero) with those measured from chondrocytes maintained in primary culture for 1, 3, and 5 days. The dots on each bar indicate individual measurements. The numbers above bars indicate the repetitions in each group. Asterisks above the lines connecting two different bars indicate a statistically significant difference. (*) denotes *p* < 0.01; (**) denotes *p* < 0.001 after a *t*-test.

Next, we aimed to determine whether TRPA1 channel expression levels in chondrocytes change when seeded in a primary culture. To investigate this, we measured pId-TRPA1 in chondrocytes at 1, 3, and 5 days after seeding and compared their average values to those of freshly dissociated chondrocytes. The results, presented as a bar graph in Figure 5, include a statistical analysis of the data.

The average pId-TRPA1 value for chondrocytes one day after seeding (−31.8 ± 1.5 pA/pF) was significantly lower (*p* < 0.01) than that of freshly dissociated chondrocytes (−42.4 ± 0.9 pA/pF). This decreasing trend continued at three days (−26.5 ± 1.5 pA/pF), which was also significantly lower than the value at one day (*p* < 0.01). However, the trend appeared to stabilize thereafter, as no significant difference was observed between the values at five days (−28.4 ± 1.6 pA/pF) and three days.

Meanwhile, as shown in the graph, the average membrane surface area at 0, 1, 3, and 5 days was 80.3 ± 1.7 μm^2^, 72.6 ± 1.9 μm^2^, 81.2 ± 1.6 μm^2^, and 86.5 ± 1.7 μm^2^, respectively. The membrane area exhibited a statistically significant reduction after one day (*p* < 0.01) compared to freshly dissociated cells, but no significant differences were observed thereafter, suggesting that membrane surface area and pId-TRPA1 are not correlated.

These findings indicate that rat articular chondrocytes undergo downregulation of TRPA1 channel expression when cultured in primary culture.

### 2.4. Incubation with Pro-Inflammatory Agents Increases pId-TRPA1

As mentioned earlier, a key reason for studying TRPA1 channels in articular chondrocytes is their potential role in inflammatory processes in cartilage. To investigate this, we examined the effects of three pro-inflammatory compounds—cytokines IL-1α and IL-1β [55] as well as lipopolysaccharide (LPS) [56]—on TRPA1 channel expression.

**Figure 6:**
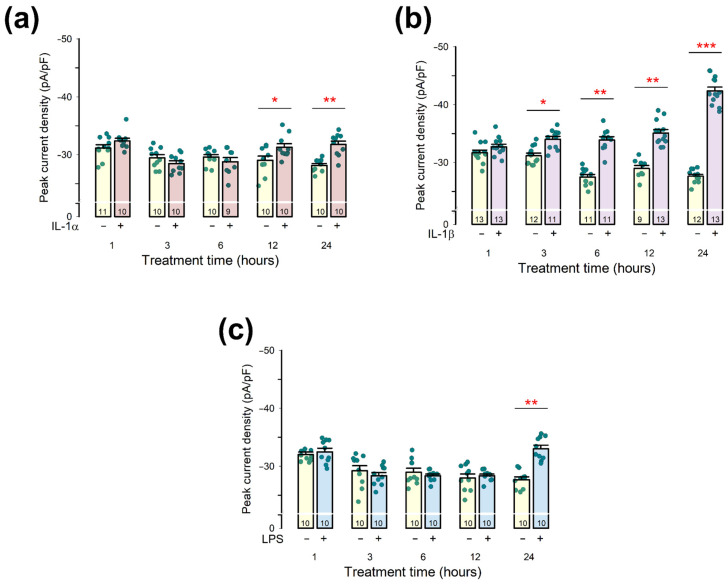
Treatment with pro-inflammatory agents induces enhancement of pId-TRPA1 in chondrocytes in primary culture. Panels (**a**–**c**) are bar graphs showing the effect of IL-1α, IL-1β, and LPS, respectively. In each of them, the average value (±S.E.) of pId-TRPA1 from chondrocytes that were either treated (+) or not (−) with the different compounds during 1, 3, 6, 12, and 24 h is compared. The dots on each bar represent individual measurements. The numbers at the bottom of each bar correspond to the sample size of each group. The asterisks above each pair of bars indicate a statistically significant difference with *p* < 0.01, *p* < 0.001, or *p* < 0.0001 for one, two, or three asterisks, correspondingly.

To assess the impact of these compounds, we performed whole-cell patch-clamp assays to measure pId-TRPA1 in primary chondrocyte cultures, comparing treated and untreated cells at 1, 3, 6, 12, and 24 h. Figure 6 presents the results for the three pro-inflammatory agents.

For IL-1α, the average pId-TRPA1 value in treated cells was significantly higher than in untreated cells at 12 h (−29.0 ± 0.7 pA/pF, n = 10 vs. −31.3 ± 0.6 pA/pF, n = 10; *p* < 0.05) and at 24 h (−28.1 ± 0.3 pA/pF, n = 10 vs. −31.8 ± 0.6 pA/pF, n = 10; *p* < 0.001).

Chondrocytes treated with IL-1β showed a statistically significant increase in the average pId-TRPA1 value compared to untreated cells at 3 h (−31.2 ± 0.4 pA/pF, n = 12 vs. −34.0 ± 0.5 pA/pF, n = 11; *p* < 0.01); 6 h (−27.5 ± 0.4 pA/pF, n = 11 vs. −33.9 ± 0.6 pA/pF, n = 11; *p* < 0.001); 12 h (−29.0 ± 0.5 pA/pF, n = 9 vs. −35.1 ± 0.6 pA/pF, n = 13; *p* < 0.001); and 24 h (−27.6 ± 0.3 pA/pF, n = 12 vs. −42.4 ± 0.7 pA/pF, n = 13; *p* < 0.0001), with the effect becoming more pronounced over time.

In contrast, LPS-treated cells exhibited a significantly higher pId-TRPA1 value only after 24 h (−27.6 ± 0.5 pA/pF, n = 10 vs. −33.0 ± 0.6 pA/pF, n = 10; *p* < 0.001).

These findings indicate that the pro-inflammatory compounds IL-1α, IL-1β, and LPS can upregulate TRPA1 channel expression in primary chondrocyte cultures, with IL-1β exerting the most pronounced effect.

### 2.5. Short-Term Exposure to IL-1β Transiently Activates I-TRPA1 in Chondrocytes

**Figure 7:**
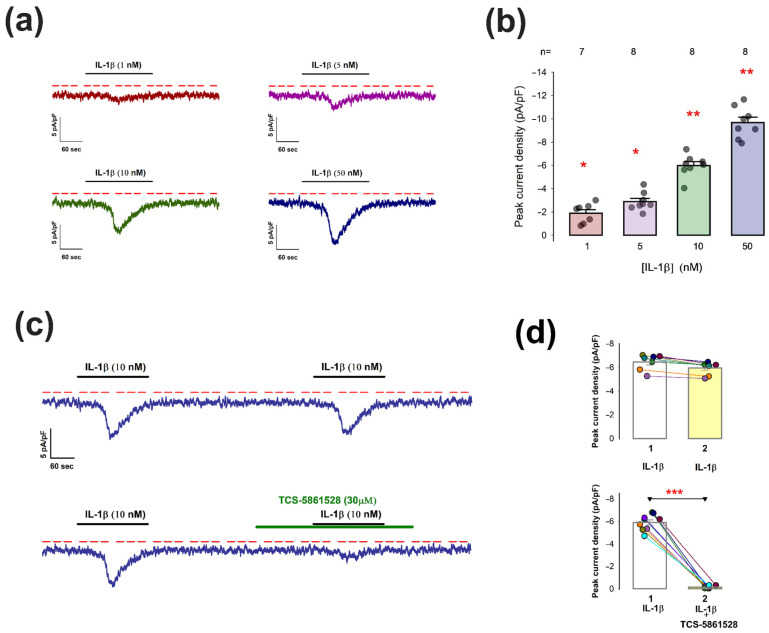
Short-term treatment of chondrocytes with IL-1β induces transient activation of I-TRPA1: (**a**) Representative traces of current density illustrating the effect of IL-1β at different concentrations. Ion current was recorded while holding Vm at −70 mV. The horizontal line at the top indicates the time in which IL-1β was administered, while the red dashed line indicates the value of zero current density. (**b**) Bar chart comparing the average (±S.E.) value of pId for each concentration of IL-1β. On each bar, the dots represent the individual measurements in different chondrocytes. The numbers above the bars indicate the number of repeats for each concentration of IL-1β, as indicated at the bottom of each bar. The asterisks above each bar indicate (*): *p* < 0.01 or (**): *p* < 0.001 of pId being significantly distinct from 0 after a z test. (**c**) Representative traces of current obtained because of tandem administering IL-1β to the extracellular medium. In the lower trace, the antagonist TCS58 was added to the extracellular medium after the first stimulus and before the second, as illustrated in the upper green bar. (**d**) Bar plots statistically comparing the average value (±S.E.) of pId of the first (1) and second pulse (2), both in the trials where IL-1β was applied only (upper) and in the one where TCS58 was administered before IL-1β in the second pulse (lower). The dots on each bar correspond to the individual measurements on the first and second pulses, the lines connecting to the dots identify the individual assays. The three asterisks above the line connecting the two bars of the lower bar graph represent a statistically significant difference (*p* < 0.0001) for a paired *t* test.

Next, to investigate whether IL-1β exerts a short-term effect on TRPA1 channel activity, we performed whole-cell patch-clamp assays using freshly dissociated chondrocytes. IL-1β was introduced into the extracellular medium, while membrane currents were continuously recorded under a voltage clamp of −70 mV. Figure 7a presents representative current traces for different IL-1β concentrations, while the bar graph in Figure 7b compares the average pId-TRPA1 values following treatment with IL-1β at 1, 5, 10, and 50 nM. As shown, IL-1β induced a transient increase in current, significantly exceeding baseline levels starting at 1 nM, with the effect magnitude increasing proportionally with IL-1β concentration. These results suggest that IL-1β has a short-term influence on TRPA1 channel activity in rat joint chondrocytes.

To confirm this, we examined whether the IL-1β-induced current transients could be inhibited by the TRPA1 antagonist TCS-5861528. First, we validated that repeated IL-1β stimulation produced consistent transient effects within the same cell. As shown in the upper trace of Figure 7c and the upper histogram of Figure 7d, a second IL-1β stimulation, applied three minutes after the first, elicited a transient current response similar in magnitude and shape to the initial response. We then tested the effect of TCS-5861528 (30 µM) on IL-1β-induced currents. After the first IL-1β stimulation triggered a transient response, TCS-5861528 was added to the extracellular medium one minute before the second stimulation. The representative trace in the lower panel of Figure 7c and the lower histogram in Figure 7d show that TCS-5861528 significantly inhibited the second IL-1β-induced current transient.

These findings demonstrate that IL-1β exerts a short-term influence on TRPA1 channel activity in rat joint chondrocytes, suggesting that it may act either directly as an agonist or indirectly through a receptor molecule located near TRPA1 channels.

### 2.6. The IL-1β-Induced Increase in pId-TRPA1 Depends on the Activation of Signaling Components Involved in Inflammation

As described previously, prolonged exposure to IL-1β (3 h or more) significantly enhanced the average peak current density induced by AITC, suggesting an upregulation of TRPA1 channel expression.

IL-1β is known to bind to the IL-1 receptor type 1 (IL-1 R1), initiating a cascade of intracellular signaling events. This process begins with the recruitment of the adaptor protein MyD88 (Myeloid Differentiation Primary Response 88), which activates downstream kinases, such as IRAKs (IL-1 receptor-associated kinases). These kinases, in turn, activate NF-κB, MAPKs (ERK, JNK, p38), and other transcription factors that drive inflammatory responses [57,58,59].

To investigate the involvement of specific signaling pathways and components in the long-term effects of IL-1β on TRPA1 channel expression, we tested several compounds known to inhibit distinct steps in the IL-1β-triggered signaling pathways. Specifically, we examined the following inhibitors: (1) IL-1 RA, a peptide mimic of MyD88 that inhibits the interaction between MyD88 and IL-1 R1 [60]; (2) IRAK1/4 inhibitor, a benzimidazole that disrupts the activity of IRAK1 and IRAK4 [61,62]; (3) PS-1145, a selective inhibitor of IκB kinase β (IKKβ) and the IKK complex [63,64]; and (4) MLN120 B, another selective inhibitor of IκB kinase β [65].

The expression of pId-TRPA1 was assessed in chondrocytes in primary culture across four experimental groups for each testing compound: (1) untreated cells serving as the control group; (2) cells exposed to IL-1β for three hours before conducting whole-cell patch-clamp assays; (3) cells treated with the compound of interest in the absence of IL-1β; and (4) cells treated with the compound of interest for one hour, followed by IL-1β treatment.

**Figure 8:**
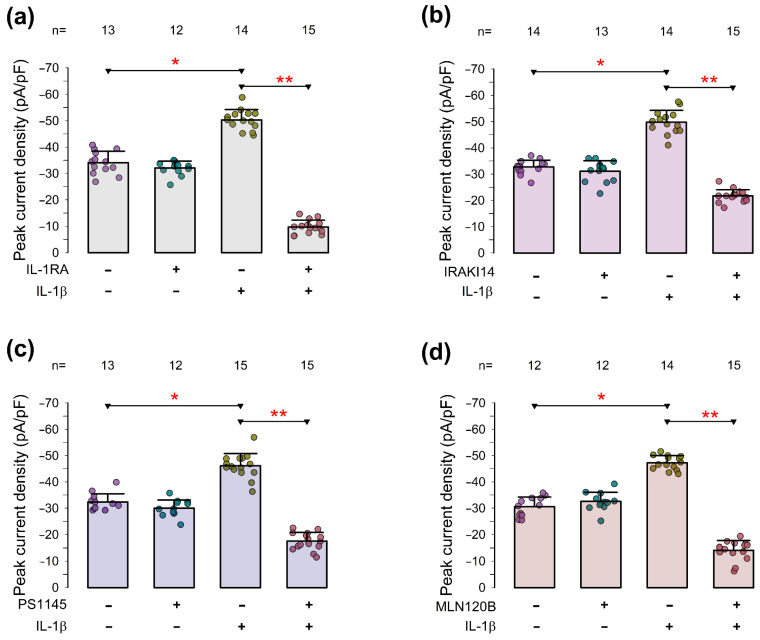
The enhancement of I-TRPA1 triggered by IL-1β relies on the activation of signaling pathways involved in the inflammatory response. Panels (**a**–**d**) show bar graphs comparing the average (±S.E.) value of pId-TRPA1 of a set of chondrocytes that were either treated (+) or not treated (−), with IL-1β, with or without pretreatment with any of the different testing compounds: IL-1 RA (**a**), IRAKI14 (**b**), PS1145 (**c**) or MLN120 B (**d**). The dots on each bar show the individual measurements of pId-TRPA1. The numbers above the bar indicate the number of repetitions of each group. The asterisks above the horizontal lines denote a statistically significant difference between the indicated groups, after a t-test. One asterisk denotes a *p* < 0.01, while two asterisks denote *p* < 0.001.

Figure 8 displays bar graphs comparing the mean values (±S.E.) of pId-TRPA1 across the four experimental groups for each compound. Figure 8a shows that the average pId-TRPA1 value in chondrocytes pre-treated with IL-1 RA and subsequently exposed to IL-1β was −9.7 ± 0.7 (n = 15) pA/pF, which is significantly lower (*p* < 0.001) than the −50.3 ± 1.0 (n = 14) pA/pF observed in chondrocytes treated exclusively with IL-1β. Similarly, Figure 8b indicates that the mean pId-TRPA1 value in chondrocytes pre-treated with IRAK1/4 and then exposed to IL-1β (−9.7 ± 0.7 pA/pF, n = 15) was significantly lower (*p* < 0.001) than the −50.3 ± 1.0 pA/pF (n = 14) recorded in chondrocytes treated solely with IL-1β. Additionally, Figure 8c shows that chondrocytes pre-treated with PS1145 and subsequently exposed to IL-1β exhibited an average pId-TRPA1 value of −17.5 ± 0.8 pA/pF (n = 15), which was significantly lower (*p* < 0.001) than the value observed in cells treated only with IL-1β (−46.1 ± 1.2 pA/pF, n = 15). Likewise, Figure 8d demonstrates that pretreatment with MLN120 B significantly reduced (*p* < 0.001) the average pId-TRPA1 value from −47.3 ± 0.7 (n = 14) to −14.1 ± 0.9 (n = 15).

These findings indicate that several components involved in the inflammation process also play a role in the signaling cascade through which IL-1β enhances TRPA1 expression. Key players in this process include IL-1 R1, the adaptor protein MyD88, and the downstream kinases IRAK1 and IRAK4.

## 3. Discussion

Articular cartilage is a smooth, resilient tissue primarily composed of water, collagen, and proteoglycans. It covers the ends of bones in synovial joints, facilitating frictionless movement and absorbing mechanical loads. As an avascular and aneural tissue, articular cartilage is maintained by chondrocytes, its only resident cell type. These cells rely on nutrient diffusion from synovial fluids for survival and the removal of metabolic waste.

Emerging evidence indicates that chondrocytes express a diverse array of ion channels [15,16]. However, the functional characterization of these channels remains incomplete, and their roles in chondrocyte physiology are poorly understood. Additionally, the mechanisms regulating these channels in both healthy and diseased states require further investigation.

Transient receptor potential (TRP) channels are widely recognized as essential mediators of various physiological processes, functioning in both neurons and non-excitable cells. While their regulatory roles in physiological and pathological contexts are becoming increasingly evident, their expression and functional significance in chondrocytes remain largely underexplored [66,67,68,69,70].

Among TRP channels, Transient Receptor Potential Ankyrin 1 (TRPA1) has garnered significant attention due to its involvement in pathological processes—most notably inflammation and pain—across various cell types and tissues [43,71,72,73,74]. Consequently, extensive research has focused on elucidating its pharmacological properties to develop therapeutic compounds targeting these conditions [40,41,75]). Nevertheless, TRPA1′s role in chondrocytes remains largely unexplored.

Studies on human osteoarthritic chondrocytes have identified the expression of nineteen distinct TRP genes, including TRPA1 and TRPM8, using next-generation sequencing (NGS) analyses [66]. Further validation of TRPA1 expression in these cells has been achieved through qRT-PCR, Western blot, and Ca^2+^-influx assays [76], confirming the presence of TRPA1 channels in osteoarthritic cartilage. However, ethical constraints limit direct comparisons between healthy and diseased human cartilage, emphasizing the need for studies that examine both healthy and osteoarthritic cartilage within the same species. Additionally, electrophysiological assays provide a more comprehensive analysis of TRPA1 channel functionality and its alterations under pathological conditions.

In this study, we used whole-cell patch-clamp electrophysiology to demonstrate that freshly isolated chondrocytes from healthy rats exhibit ionic currents with biophysical and pharmacological properties consistent with TRPA1 channels. These currents display hallmark features observed in other cell types, including outward rectification, a near-zero reversal potential (indicative of poor cation selectivity) [77], and sensitivity to AITC as well as the specific agonists PF-4840154 and JT010 [78,79]. Moreover, these currents were potently inhibited by the TRPA1 antagonists HC-030031 [48] and TCS-5861528 [51], providing strong functional evidence for TRPA1 channel expression in native rat joint chondrocytes. The significance of these findings lies in demonstrating that TRPA1 channels are expressed in both healthy and osteoarthritic chondrocytes, suggesting a broader physiological role beyond pathological conditions.

Additionally, we found that TRPA1-mediated ionic currents in freshly dissociated chondrocytes are size-dependent, with medium-sized chondrocytes exhibiting the strongest TRPA1 channel activity. This observation suggests a functional specialization of TRPA1 across cartilage zones, potentially influencing mechanochemical signaling in distinct cartilage layers. Such stratification may reflect adaptations to mechanical stress or biochemical gradients within the tissue.

Furthermore, we observed a progressive decline in TRPA1-mediated current density in chondrocytes maintained in primary culture over time. This temporal reduction suggests that TRPA1 channel activity is dynamically downregulated under ex vivo culture conditions, likely reflecting phenotypic adaptation to an altered microenvironment. These findings align with recent studies linking TRPA1 expression to extracellular matrix (ECM) stiffness [80], a parameter known to shift in culture systems. Given TRPA1′s established role as a mechanosensitive transducer in various cell types [81,82], its sensitivity to ECM-derived mechanical cues may underlie this regulatory mechanism. This suggests that TRPA1 plays a dual role in chondrocyte physiology, integrating inflammatory and biomechanical pathways during osteoarthritis progression.

Moreover, our study demonstrates that long-term (days) exposure to pro-inflammatory agents (IL-1α, IL-1β, and LPS) enhances TRPA1 current density, suggesting upregulation of TRPA1 channel expression. These results are consistent with previous studies using calcium influx measurements and Western blot assays, which showed that IL-1β upregulates TRPA1 expression and activity in rat chondrocytes [83]. Additionally, using specific inhibitors, we identified that IL-1β-driven TRPA1 upregulation involves key inflammatory signaling components, including IL-1 R1 and downstream kinases IRAK1/4 and MAPKs.

Finally, we demonstrate that IL-1β treatment of freshly dissociated chondrocytes not only induces a long-term increase in TRPA1 current density but also triggers immediate transient currents with characteristics like those produced by TRPA1 agonists. These transient currents are significantly inhibited by the TRPA1 antagonist TCS-5861258, supporting a functional link between IL-1β and TRPA1 activation. Notably, similar findings have been reported in nodose ganglion sensory neurons, where IL-1β induces transient calcium influxes that can be inhibited by TRPA1 antagonists [84].

While the rapid response suggests a potential direct interaction between IL-1β and TRPA1 channel subunits, indirect activation via adjacent membrane components cannot be excluded. Supporting this mechanism, TRPA1 channels are known to associate with TRPV4 channels, and recent studies have identified novel TRPV4 inhibitors that concurrently suppress TRPA1 activity—a dual-target strategy with therapeutic potential for pain and inflammation [85]. This functional interplay suggests that IL-1β’s effects may involve cross talking between TRPA1 and neighboring membrane proteins. However, further investigation is needed to delineate direct vs. indirect pathways.

Overall, our findings reveal a previously unrecognized role of TRPA1 in healthy chondrocytes, expanding their functional significance beyond disease states. These results lay the groundwork for future studies exploring the mechanistic pathways linking TRPA1 to inflammatory and mechanical stimuli in cartilage homeostasis and osteoarthritis progression.

### Future Perspectives

Future research should focus on validating these findings in osteoarthritis (OA) models and investigating the long-term effects of TRPA1 modulation on cartilage health and OA progression. Additionally, exploring the interplay between TRPA1 activation, mechano transduction, and inflammation could reveal novel pathways for therapeutic intervention. Developing dual TRPA1/TRPV4 inhibitors and strategies targeting matrix stiffness-dependent TRPA1 activation holds promise for more effective OA treatments.

## 4. Materials and Methods

### 4.1. Isolation of Freshly Dissociated Cells and Establishment as a Primary Culture

Articular chondrocytes were cultured following previously described protocols [34,86,87]. Minced hip condyles were treated with 0.2% type II collagenase in PBS for 2–3 h at 37 °C. After enzymatic digestion, the chondrocytes were mechanically dissociated by gentle pipetting, followed by washing and centrifugation twice. The cells were then resuspended in DMEM supplemented with 10% calf serum. Freshly isolated chondrocytes were seeded onto glass coverslips at a density of 1–3 × 10^5^ cells/mL and maintained under sterile conditions at 37 °C in a humidified atmosphere with 5% CO_2_ until further use.

### 4.2. Recording of Ion Currents Using the Whole-Cell Patch-Clamp Technique

Ion currents were measured using the whole-cell patch-clamp method, following established protocols detailed elsewhere [84,85]. Coverslips containing articular chondrocytes were placed in a recording chamber mounted on the stage of an inverted microscope (Diaphot 300, Nikon, Tokyo, Japan). The chamber was filled with an bath solution (composition described in Section 4.4) and maintained under continuous perfusion. A U-shaped glass tubing filled with 2% agarose in 500 mmol/L KCl was used to ensure electrical contact between the bathing solution and the reference electrode. The reference electrode was positioned in a chamber containing 500 mmol/L KCl. Micropipettes were prepared from borosilicate glass tubing (cat. 34500-99, Kimble Chase, Vineland, NJ, USA) using a horizontal puller (P-87, Sutter Instrument Co., Novato, CA, USA), resulting in a tip resistance of 2–5 MΩ. These pipettes were backfilled with a pipette solution (as described in Section 4.4) and attached to a pipette holder connected to a piezoelectric-driven micromanipulator (PCS6000, Burleigh Co., Bismarck, ND, USA). The pipettes were maneuvered to contact the cells, and patch rupture was initiated by applying suction once a gigaseal exceeding 2 GΩ (typically 5 GΩ) was achieved.

### 4.3. Measurement of Membrane Capacitance

Capacitive transients were induced by applying hyperpolarizing square voltage pulses ranging from −100 to −110 mV and recorded at a sampling rate of 10 kHz. Membrane capacitance was then estimated offline by integrating the area under the capacitive transient curve at the onset of the pulse. The integrated value was divided by the pulse amplitude (−10 mV) using the following formula:

$$Cm=\frac{\int_{t0}^{\infty }Ic\cdot ∆t}{∆V}$$

where *Cm* represents membrane capacitance, *Ic* denotes capacitive current, and Δ*V* signifies the pulse amplitude (−10 mV).

The estimate of the integral was performed using the Clampfit module of pClamp 8.0 (Molecular Devices, San Jose, CA, USA). The cell membrane area was further determined, assuming a specific capacitance of 1 µF/cm^2^ [88].

### 4.4. Chemicals and Solutions

The AITC (Cat. 377430) was purchased from MERCK, St. Louis, MO, USA. All other chemicals were acquired from Cayman Chemicals, including agonists JT010 (Cat. 29732), PF-4840154 (Cat.28915); antagonists TCS-5861528 (Cat.18380), HC-030031 (Cat.11923), and signaling blockers IL-1 R antagonist (Cat.21349), IRAK1/4 inhibitor (Cat.17540), PS-1145 (Cat.14862), and MLN120 B (Cat.32819), whose testing concentrations were 50 µM, 5 µM, 500 nM and 100 nM respectively.

The composition of the pipette solution was (mM): 140 Cs-Gluc (Cesium Gluconate), 10 EGTA, 10 HEPES, and 2 Mg-ATP. The pH was adjusted to 7.4 with CsOH, and the osmolarity was 300 mOsm. The composition of the bath solution was (mM): 140 NaCl, 5 KCl, 2 CaCl_2_, 1 MgCl_2_, and 10 HEPES. The pH was adjusted to 7.4 with NaOH, and the osmolarity was adjusted to 310 mOsm by d-glucose.

### 4.5. Analysis of Data

#### 4.5.1. Fitting of Data to Estimate EC50 Values

The average pId-TRPA1 values were adjusted to the following four-parameter sigmoidal equation:

$${pId}_{x}={pId}_{0}+\frac{a}{\left(1+10^{\left(\frac{-\left(x-x_{0}\right)}{b}\right)}\right)}$$

where ${pId}_{x}$ is the calculated peak current density, *x* is log_10_ of a given testing concentration (μM), *a*, *b*, *pId*_0_, and *x*_0_ are adjustment parameters.

Therefore,

$${EC}_{50}=10^{x_{0}}$$

Numerical curve fitting was made with the analysis module of Sigmaplot for Windows, version 15.

#### 4.5.2. Statistical Analysis

Statistical tests were conducted using the statistical analysis modules in EXCEL (Microsoft 365) and the tools available in Sigmaplot for Windows, version 15. For experiments involving more than two groups, one-way ANOVA was performed, followed by multiple comparisons against the control group. Initially, equal variance was assessed using the Brown–Forsythe test; if this test failed, a nonparametric approach (Kruskal–Wallis one-way ANOVA on ranks) was applied, followed by multiple comparisons versus the control group using Dunnett’s method. For comparisons involving only two groups, a *t*-test was employed. A significance level of *p* < 0.01 was used as the threshold for rejecting the null hypothesis.

## 5. Conclusions

This study provides strong evidence supporting TRPA1’s role in chondrocyte function and inflammation, highlighting its potential as a novel target for OA treatment. By characterizing the biophysical and pharmacological properties of ion currents linked to TRPA1 expression and their regulation by inflammatory signals, we lay the groundwork for new therapeutic strategies to combat OA and other cartilage-degenerative diseases.

## Data Availability

Data are contained within the article.
